# Screening and Identification of Key Biomarkers of Gastric Cancer: Three Genes Jointly Predict Gastric Cancer

**DOI:** 10.3389/fonc.2021.591893

**Published:** 2021-08-17

**Authors:** Meng-jie Shan, Ling-bing Meng, Peng Guo, Yuan-meng Zhang, Dexian Kong, Ya-bin Liu

**Affiliations:** ^1^Department of Plastic Surgery, Peking Union Medical College Hospital, Beijing, China; ^2^Chinese Academy of Medical Sciences & Peking Union Medical College, Beijing, China; ^3^Cardiology Department, Beijing Hospital, National Center of Gerontology, Beijing, China; ^4^Department of Orthopedics, The Fourth Hospital of Hebei Medical University, Shijiazhuang, China; ^5^Department of Cardiology, The Third Medical Centre of Chinese PLA General Hospital, Beijing, China; ^6^Department of Endocrinology, The Fourth Affiliated Hospital of Hebei Medical University, Shijiazhuang, China; ^7^Department of General Surgery, The Fourth Affiliated Hospital of Hebei Medical University, Shijiazhuang, China

**Keywords:** gastric cancer, gene expression profiling, bioinformatics analysis, weighted gene co-expression network analysis, neural network model

## Abstract

**Background:**

Gastric cancer (GC) is one of the most common cancers all over the world, causing high mortality. Gastric cancer screening is one of the effective strategies used to reduce mortality. We expect that good biomarkers can be discovered to diagnose and treat gastric cancer as early as possible.

**Methods:**

We download four gene expression profiling datasets of gastric cancer (GSE118916, GSE54129, GSE103236, GSE112369), which were obtained from the Gene Expression Omnibus (GEO) database. The differentially expressed genes (DEGs) between gastric cancer and adjacent normal tissues were detected to explore biomarkers that may play an important role in gastric cancer. Gene Ontology (GO) and Kyoto Encyclopedia of Genes and Genomes (KEGG) analyses of overlap genes were conducted by the Metascape online database; the protein-protein interaction (PPI) network was constructed by the STRING online database, and we screened the hub genes of the PPI network using the Cytoscape software. The survival curve analysis was conducted by km-plotter and the stage plots of hub genes were created by the GEPIA online database. PCR, WB, and immunohistochemistry were used to verify the expression of hub genes. A neural network model was established to quantify the predictors of gastric cancer.

**Results:**

The relative expression level of cadherin-3 (CDH3), lymphoid enhancer-binding factor 1 (LEF1), and matrix metallopeptidase 7 (MMP7) were significantly higher in gastric samples, compared with the normal groups (p<0.05). Receiver operator characteristic (ROC) curves were constructed to determine the effect of the three genes’ expression on gastric cancer, and the AUC was used to determine the degree of confidence: CDH3 (AUC = 0.800, P<0.05, 95% CI =0.857-0.895), LEF1 (AUC=0.620, P<0.05, 95%CI=0.632-0.714), and MMP7 (AUC=0.914, P<0.05, 95%CI=0.714-0.947). The high-risk warning indicator of gastric cancer contained 8<CDH3<15 and 10<expression of LEF1<16.

**Conclusions:**

CDH3, LEF1, and MMP7 can be used as candidate biomarkers to construct a neural network model from hub genes, which may be helpful for the early diagnosis of gastric cancer.

## Background

Gastric cancer (GC) is one of the most common cancers all over the world and causes high mortality. Especially in China, where almost a third of the world’s new cases of GC occur (1). The treatment of gastric cancer has limited effect. Although the survival of some patients with advanced gastric cancer can be prolonged through chemotherapy, most chemotherapy has limited efficacy and a short maintenance time. The 2-year survival rate is less than 10% (2). The rapid development of tumor transcriptome data based on the second-generation high-throughput sequencing technology comprehensively reveals the multi-genetic and highly heterogeneous measuring points of the tumor. The diagnosis of the tumor molecular and targeted treatment is an effective means for improving the early diagnosis rate (3). It is also the direction and goal of the development of the clinical treatment of the tumor, and the achievement of this goal will ultimately depend on the search for specific tumor biomarkers (4).

Detection through serum tumor markers is a noninvasive diagnostic method commonly used in the clinic. However, conventional assays for carcinoembryonic antigen (CEA) and carbohydrate antigen 19-9 (CA19-9) are not specific or sensitive enough for accurate diagnosis of GC, and it is necessary to develop some novel biomarkers (5). Bioinformatics technology has been increasingly used to authenticate the differentially expressed genes (DEGs) and underlying pathways that are related to the occurrence and progression of GC, which can help researchers excavate the potential genetic targets of diseases (6).

However, it is difficult to obtain credible results when using the independent microarray technology because of the higher false-positive rates (7). Therefore, this study reanalyzed four expression profiling datasets downloaded from the Gene Expression Omnibus (GEO) dataset. T1he DEGs were searched for by GEO2R tools. The Kyoto Encyclopedia of Genes and Genomes (KEGG) and Gene Ontology (GO) analyses of overlap genes among four datasets were conducted in the Metascape database. The molecular mechanisms of the occurrence and progression of gastric cancer were detected by enrichment analysis of functions and pathways and protein-protein interaction (PPI) network analysis. The hub genes were analyzed by Cytoscape. The survival curve analysis was performed by km-plotter and the stage plots of hub genes were generated by the GEPIA online database. CDH3, LEF1, and MMP7 were used as candidate biomarkers to construct a back propagation neural network model.

## Methods

### Downloaded Public Data

The GEO (8) contains a variety of high-throughput sequencing experimental data. Four expression profiling datasets [GSE118916 (9), GSE54129 (10), GSE103236 (11), and GSE112369 (12)], which all screen genes associated with the formation of gastric cancer from normal tissue, were downloaded from the GEO human gene expression array.

### Intra-Group Data Repeatability Test

The statistical analysis and graphic drawing were performed *via* the R programming language. The correlation between all samples from the same dataset was tested using a correlation heat map, which was constructed in R. The method of principal component analysis was used to analyze the dimensionality of the data and observe the distribution of the data (13).

### Identification of DEGs

GEO2R (8) was used to identify DEG between gastric cancer and normal tissues adjacent to the cancer. We set the threshold as logFC≥1 or ≤-1, P value < 0.05. A volcano mapping tool (https://shengxin.ren) was used to map the volcano. Four datasets were then introduced into Fun Rich (a feature rich analysis tool) (http://www.funrich.org/) to filter DEGs. The DEGs shared between the four datasets were obtained *via* the Venn diagram, which was depicted by the Venn tool. The Circos diagram shows gene overlap and function overlap between different gene lists.

### Establishing Weighted Gene Co-Expression Network Analysis (WGCNA)

The raw data of GSE54129 were preprocessed by the R package (version 3.5.0). The control group and gastric cancer samples were arranged according to the P value from small to large. A total of 5000 DEGs were selected for WGCNA (14). WCGNA can identify highly synergistically changing sets of genes. Gene networks conform to a scale-free distribution. According to this point, the gene network can be divided into different modules based on the similarity of expression to find the pivot genes. WGCNA performed module identification. Then, WCGNA established a gene co-expression network and we studied the module relationship. Finally, modules were associated with clinical characteristics.

### Visual Analysis of Gene Expression Networks

DAVID (15) was used for function and pathway enrichment analysis of differential genes. Metascape (16) was again used for DEG enrichment analysis. STRING (17) was used to construct the protein-protein interaction network. Gene Set Enrichment Analysis (18) (GSEA) is an analysis method for genome-wide expression profile microarray data that compares genes with predefined gene sets. That is, based on the existing information base of gene location, properties, functions, biological significance, etc., a molecular tag database is constructed. In this database, known genes are classified according to chromosomal positions, established gene sets, patterns, and tumor-related genes. The group and GO gene set is used to group and classify multiple functional gene sets. By analyzing the gene expression profile data, we can understand their expression status in a specific functional gene set, and whether this expression status has some statistical significance. After analyzing all sequenced genes of gastric cancer tissue and normal gastric tissue by GSEA software and importing gene annotation files, the software analyzed the expression networks between genes. We therefore fully understand the effect of the richness of the feature set on the biological function of genes.

### RT-qPCR

CDH3, MMP7, and LEF1-specific primers of human and mouse were designed (**Table 1**). Gastric cancer and normal gastric tissue samples were used to extract total RNA. Then, mDNA was used as the template and cDNA was transcribed with random primers (HiScript III 1st Strand cDNA Synthesis Kit, Beijing, China). Then a qPCR fluorescence kit (Taq Pro Universal SYBR qPCR Master Mix, Beijing, China) was used to quantitatively analyze the expression of the target gene. 2-ΔΔCt was expressed as a fold change in gene expression relative to the experimental group compared to the control. GAPDH was used as an internal reference.

**Table 1:** Primers and their sequences for PCR analysis.

Primer	Sequence (5′–3′)
**Human**	
GAPDH-F	CGGAGTCAACGGATTTGGTCGTA
GAPDH-R	AGCCTTCTCCATGGTGGTGAAGAC
CDH3-F	ATCATCGTGACCGACCAGAAT
CDH3-R	GACTCCCTCTAAGACACTCCC
MMP7-F	ATGTGGAGTGCCAGATGTTGC
MMP7-R	AGCAGTTCCCCATACAACTTTC
LEF1-F	CCCGTGAAGAGCAGGCTAAA
LEF1-R	AGGCAGCTGTCATTCTTGGA
**Mouse**	
CDH3-F	AGTGTTCTGGAGGGAGTAATGC
CDH3-R	CCACCACCCCATTGTAAGTG
MMP7-F	CTTACCTCGGATCGTAGTGGA
MMP7-R	CCCCAACTAACCCTCTTGAAGT
LEF1-F	GCCACCGATGAGATGATCCC
LEF1-R	TTGATGTCGGCTAAGTCGCC

### Western Blot Analysis

Gastric cancer tissue was stored at -196°C. For the MMP7 protein, the anti-MMP7 polyclonal antibody (1: 500 dilution, Proteintech, USA) was used. CDH3 polyclonal antibody (dilution rate = 1:500, Protientech, USA) was used to detect CDH3. LEF1 polyclonal antibody was used to detect LEF1 (dilution rate = 1:500, Protientech, USA). The result was analyzed with Image-Pro Plus.

### Animal Models

Ten BALB/c-nu-nu nude mice were used to construct a gastric cancer mouse model. MGC-803 cells were injected into the gastric serosal layer of nude mice to establish the model of gastric tumor in situ. The cultured cells were resuspended to the concentration of 1.0×10^8/ml with a 1:1 mixture of matrix adhesive.

### Immunohistochemistry

The expression of MMP7, CDH3, and LEF1 in gastric tissues was analyzed by immunohistochemistry. After routine sectioning, the slides were dewaxed, dehydrated by gradient alcohol, blocked and inactivated by endogenous peroxidase, repaired by an antigen, and blocked by goat serum. Primary antibody was added and the mixture was incubated at 4°C. Labeled secondary antibody was added and the mixture was incubated at 37°C. Horseradish peroxidase labeling solution was added, the mixture was stained with DAB/H2O2, counterstained with hematoxylin, conventionally dehydrated, made transparent, and observed with a microscope after mounting.

### Neural Network Model

We randomly divided the data into a training set and validation set, with a ratio of 1:7. We set 35 samples as the training set and 5 samples as the validation set. We used Matlab (version 10) for machine learning, trained 2678 steps, and established a predictive model when the true value was close to the predicted value. The output variable was the relative expression of MMP7. The training error was 0.033031, and the R value was 0.9624.

### Statistical Analysis

The unpaired Student’s t-test was used to compare the two sets of data to determine statistical significance. Pearson’s test was used to compare the correlation between gene expression and gastric cancer. Receiver operator characteristic (ROC) curve analysis was performed to determine the usefulness of MMP7, CDH3, and LEF1 in predicting gastric cancer. The high-risk early warning range of gastric cancer was analyzed by the cubic spline interpolation algorithm. The Pearson-rho test was used to calculate the expression of hub genes and status of GEA for the correlation analysis.

## Results

### Checking Data Quality

**Figure 1:**
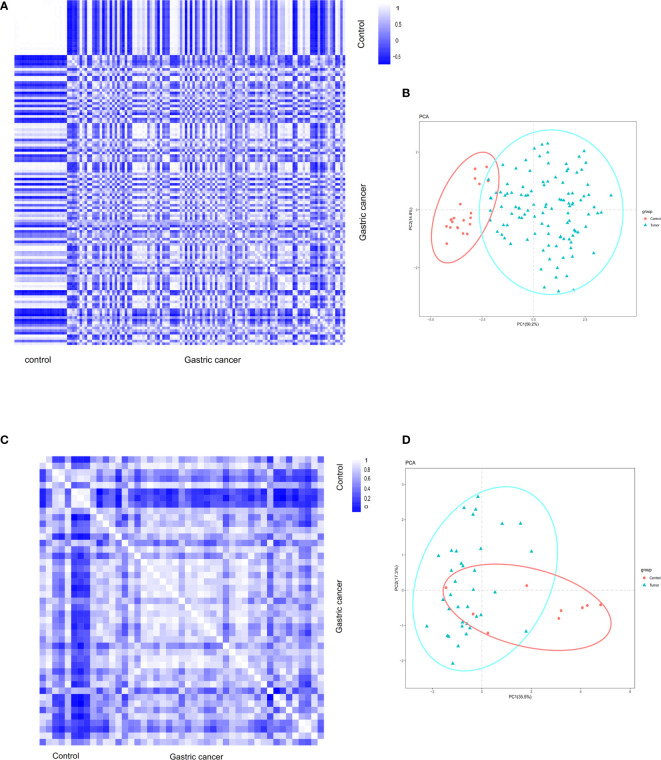
**(A)** Pearson’s correlation analysis of samples from the GSE54129 dataset. The color reflects the intensity of the correlation. When 0<correlation<1, there exists a positive correlation. When -1<correlation<0, there exists a negative correlation. The larger the absolute value of a number the stronger the correlation. **(B)** PCA of samples from the GSE54129 dataset. In the figure, principal component 1 (PC1) and principal component 2 (PC2) are used as the X-axis and Y-axis, respectively, to draw the scatter diagram, where each point represents a sample. In such a PCA diagram, the farther the two samples are from each other, the greater the difference is between the two samples in gene expression patterns. **(C)** Pearson’s correlation analysis of samples from the GSE112369 dataset. The color reflects the intensity of the correlation. **(D)** PCA of samples from the GSE112369 dataset.

Pearson’s correlation test and the analysis of principal component analysis (PCA) were used to verify the distribution of data within a group. Based on Pearson’s correlation test, the data within the group were well distributed in the gastric cancer group and the control group in the GSE54129 dataset (**Figure 1A**). Based on PCA analysis, the repeatability of the data in the GSE54129 group also met the analysis requirements (**Figure 1B**). According to Pearson’s correlation test, the strong correlation between the GSE112369 in each group was shown (**Figure 1C**). PCA showed that the GSE112369 data set was well distributed (**Figure 1D**).

### The Identification of DEGs

**Figure 2:**
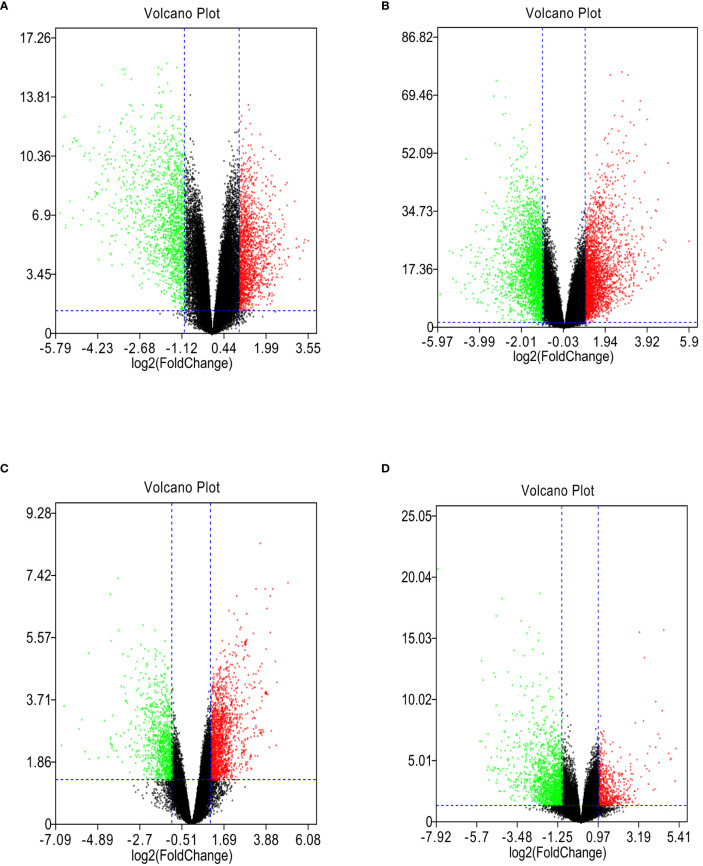
**(A)** The volcano plot illustrates the differences between control and gastric cancer tissues after analysis of the GSE118916 dataset with GEO2R. **(B)** The volcano plot illustrates the difference between control and gastric cancer tissues after analysis of the GSE54129 dataset with GEO2R. **(C)** The volcano plot illustrates the difference between control and gastric cancer tissues after analysis of the GSE103236 dataset with GEO2R. **(D)** The volcano plot illustrates the difference between control and gastric cancer tissues after analysis of the GSE112369 dataset with GEO2R.

**Figure 3:**
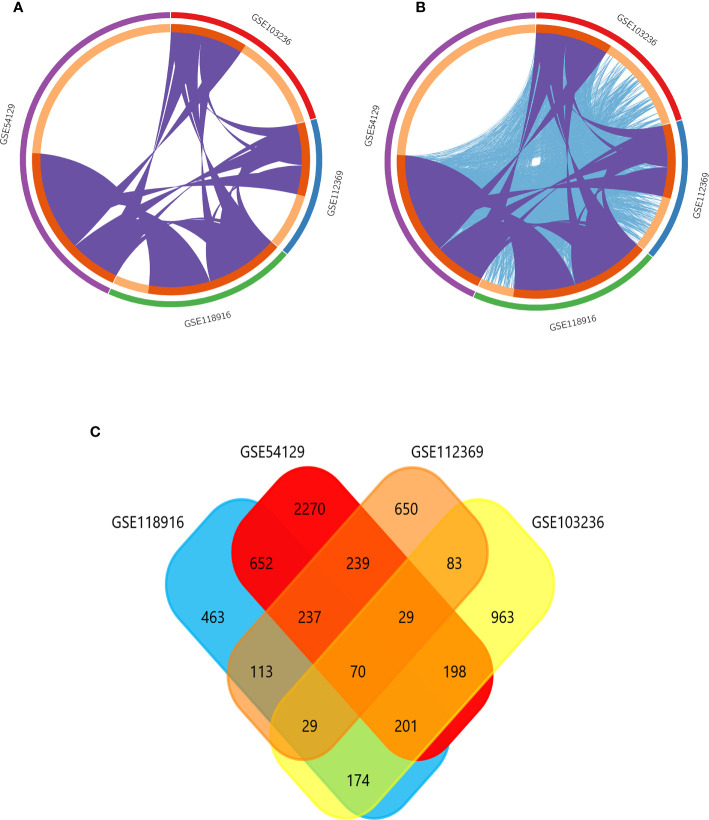
**(A)** Overlap between differently expressed gene lists of GSE118916, GSE54129, GSE103236, and GSE112369 only at the gene level, where purple curves link identical genes; **(B)** overlap between differently expressed gene lists of GSE118916, GSE54129, GSE103236, and GSE112369 not only at the gene level, but also at the shared term level, where blue curves link genes that belong to the same enriched ontology term. The inner circle represents gene lists, where hits are arranged along the arc. Genes that hit multiple lists are colored in dark orange, and genes unique to a list are shown in light orange. **(C)** The Venn diagram demonstrates that 70 genes were contained in the GSE118916, GSE54129, GSE103236, and GSE112369 datasets simultaneously.

GSE118916, GSE54129, GSE103236, and GSE112369 datasets were used to draw a volcano map (**Figures 2A–D**). Circos analysis was used to find that GSE54129 and GSE112369 had an overlap of DEGs (**Figure 3A**). Concurrently, there was also an overlap in the function of genes (**Figure 3B**). The threshold was set as logFC≥1 or ≤-1, P value < 0.05. These results have shown that a total of 3231 DEGs in the GSE118916 dataset, 5273 DEGs in the GSE54129, a total of 2557 DEGs in the GSE103236, and a total of 2477 DEGs in the GSE112369 dataset were identified. Seventy genes overlapped in the four datasets using the analysis of the Venn diagram (**Figure 3C**).

### Construction of Co-Expression Modules by Weighted Gene Co-Expression Network Analysis (WGCNA)

**Figure 4:**
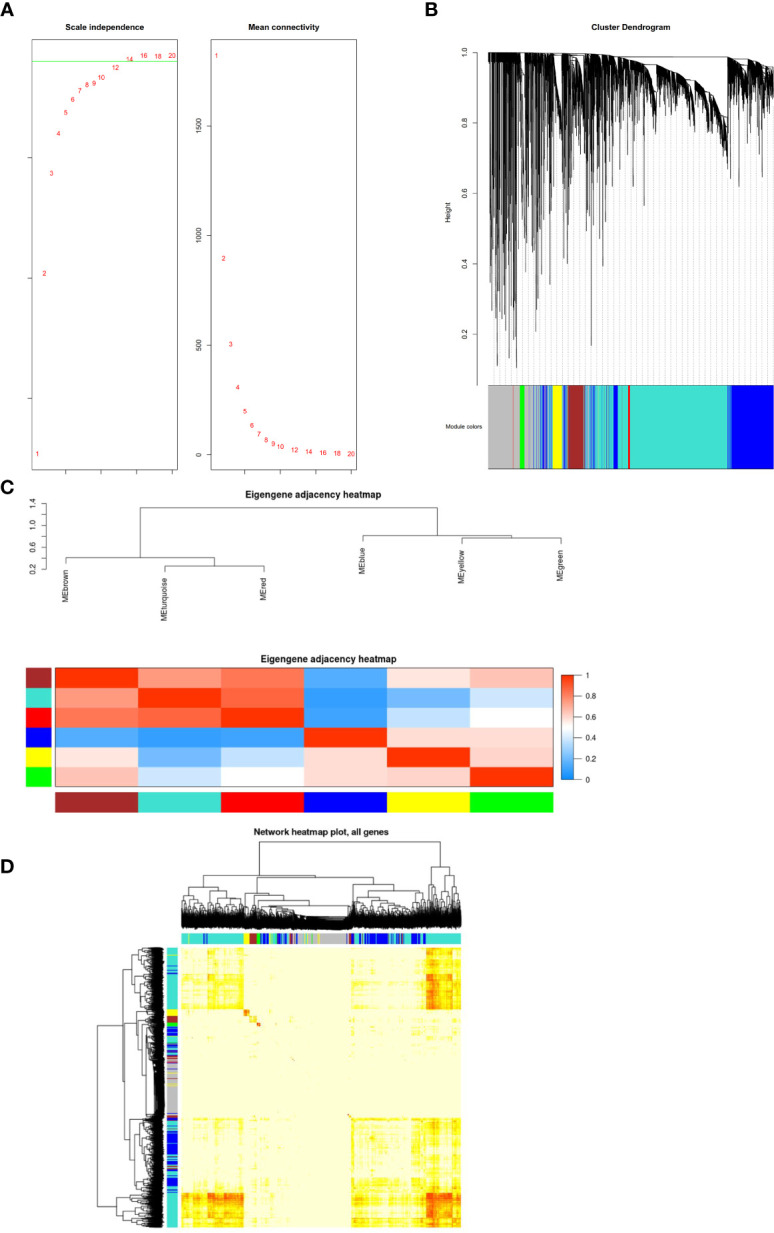
**(A)** Construction of co-expression modules by the weighted gene co-expression network analysis (WGCNA) package in R. The default value of the parameter beta is 1 to 30. The horizontal axis of the graph above represents the weight parameter β, and the vertical axis of the left graph represents the square of the correlation coefficient between log (k) and log [p (k)] in the corresponding network. The higher the square of the correlation coefficient, the more the network approaches the distribution without network scale. The vertical axis of the right figure represents the mean of the adjacent function of all genes in the corresponding gene module. **(B)** The cluster dendrogram of genes in GSE54129. Each branch in the figure represents one gene, and every color below represents one co-expression module. **(C)** Clustering visualization of samples. Heatmap plot of the adjacencies in the hub gene network. Heatmap of the correlation between module eigengenes and the disease of gastric cancer. The red module was the most positively correlated with status, and the blue module was the most negatively correlated with status. **(D)** Interaction relationship analysis of co-expression genes. Different colors of horizontal axis and vertical axis represent different modules. The brightness of yellow in the middle represents the degree of connectivity of different modules. There was no significant difference in interactions among different modules, indicating a high-scale independence degree among these modules.

The GSE54129 datasets were used for WCGNA network analysis. We set the power value as 12 (**Figure 4A**). To generate important functional modules, the parameter was set as 0.2 (**Figure 4B**). Different modules were found to have cooperative or antagonistic relationships (**Figure 4C**). The redder the module, the more likely it is to develop gastric cancer, and the bluer the module, the less likely it is to develop gastric cancer (**Figures 4C, D**).

### Functional Annotation

**Figure 5:**
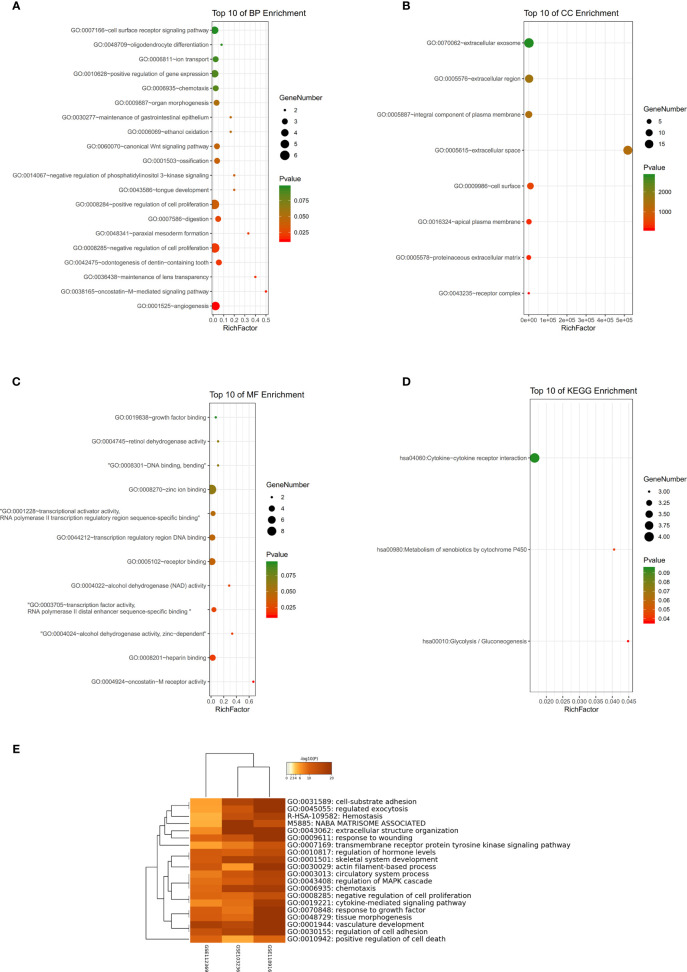
**(A–C)** Detailed information relating to changes in the biological processes (BP), cellular components (CC), and molecular functions (MF) of DEGs in gastric cancer and control tissues through the GO enrichment analyses. **(D)** The KEGG pathway analysis of DEGs. KEGG, Kyoto Encyclopedia of Genes and Genomes; GO, Gene Ontology; DEGs, differentially expressed genes. **(E)** Histogram of enriched terms across input differently expressed gene lists, colored by p-values, *via* Metascape.

GSE54129 datasets were used for GO and KEGG analyses. Biological processes (BP) of GO analysis showed that there were variations including angiogenesis, oncostatin-M-mediated signaling pathway, and so on (**Figure 5A**). The analysis of cell components (CC) showed major variations including the extracellular space, apical plasma membrane, and the cell surface (**Figure 5B**). The analysis of molecular function (MF) variations included oncostatin-M receptor activity, heparin binding, alcohol dehydrogenase activity, and zinc-dependent (**Figure 5C**). In the analysis of KEGG, genes were found to be mainly enriched in glycolysis/gluconeogenesis, metabolism of xenobiotics by cytochrome P450, and cytokine-cytokine receptor interaction (**Figure 5D**). The results of GO enrichment are shown in **Figure 5E**.

**Figure 6:**
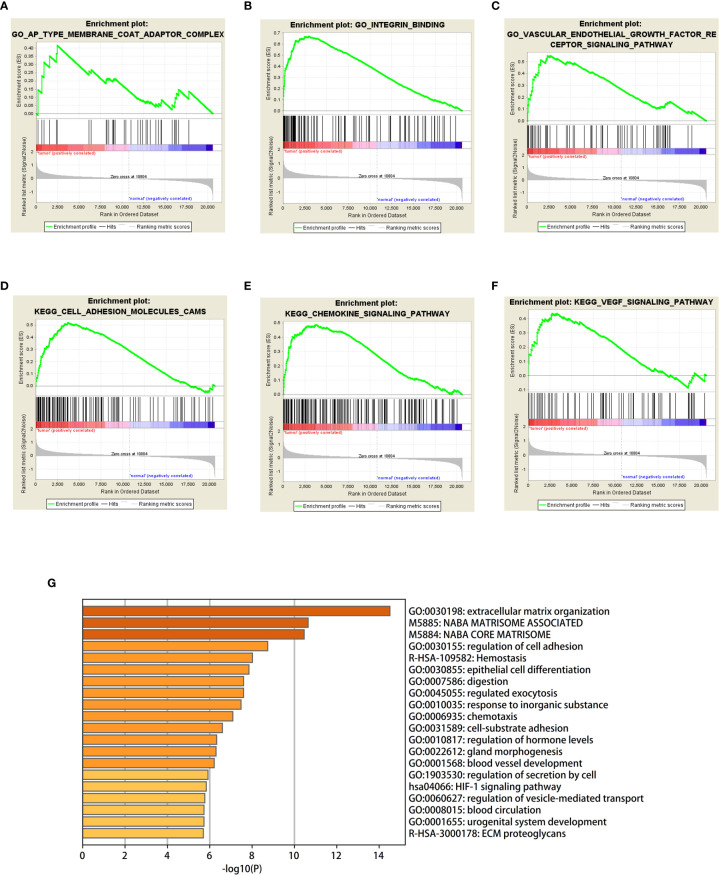
**(A–F)** Based on gene set enrichment analysis (GSEA), the main GO term and signaling pathway of differentially enriched genes in gastric cancer tissues and normal tissues were investigated. **(G)** Bar graph of enriched terms across input gene lists, colored by p-values.

GSEA was used to detect the functional enrichment and pathway analysis of DEGs (**Figures 6A–F**). The analysis of GO of gastric cancer (111 samples) showed that 2992/4374 genes were upregulated. When the threshold was set as p value < 0.05, 326 genes were identified. When the threshold was set as p value <0.01, 31 genes were identified. In addition, the analysis of GO of normal tissue (21 samples) showed that 1382/4374 gene sets were downregulated. When the threshold was set as p value < 0.05, 32 genes were identified, and when the threshold was set as p value < 0.01, 4 genes were identified. **Table 2** lists the upregulated and downregulated genes. GSEA also revealed upregulated gene sets in gastric cancer, of which 99/176 genes were upregulated in gastric cancer compared with control. When the threshold was set as p value <0.05, the result showed that nine genes were enriched. When the threshold was set as p value <0.01, one gene set was enriched significantly in control. In total, 77/176 genes were downregulated in gastric cancer, and 2 gene sets were enriched when p value <0.05. The nine important gene sets correlated with gastric cancer are displayed in **Table 3** according to NES, including

**Table 2:** Functional enrichment analysis of DEGs in gastric cancer using GSEA.

Gene set name	SIZE	ES	NES	Rank at max
**Upregulated**				
GO_BLASTOCYST_FORMATION	25	0.63	1.73	2999
GO_POSITIVE_REGULATION_OF_PHOSPHOPROTEIN_PHOSPHATASE_ACTIVITY	15	0.76	1.72	2572
GO_CLATHRIN_ADAPTOR_COMPLEX	25	0.46	1.70	2433
GO_AP_TYPE_MEMBRANE_COAT_ADAPTOR_COMPLEX	35	0.41	1.68	2433
GO_NEGATIVE_REGULATION_OF_CELL_KILLING	18	0.76	1.68	1579
GO_REGULATION_OF_PROTEIN_MATURATION	73	0.59	1.66	2195
**Downregulated**				
GO_DEACETYLASE_ACTIVITY	49	-0.60	-1.85	4264
GO_PROTEIN_DEACETYLASE_ACTIVITY	40	-0.57	-1.80	4264
GO_MITOCHONDRIAL_RESPIRATORY_CHAIN_COMPLEX_ASSEMBLY	57	-0.54	-1.72	8334
GO_HYDROLASE_ACTIVITY_ACTING_ON_CARBON_NITROGEN_BUT_NOT_PEPTIDE_BONDS_IN_LINEAR_AMIDES	73	-0.45	-1.65	4911
GO_NUCLEOSIDE_BISPHOSPHATE_BIOSYNTHETIC_PROCESS	17	-0.61	-1.63	3192
GO_FATTY_ACYL_COA_BINDING	29	-0.62	-1.63	4667

ES, enrichment score; NES, normalized enrichment score.

**Table 3:** Pathway enrichment analysis of DEGs in gastric cancer using GSEA.

Gene set name	SIZE	ES	NES	Rank at max
**Upregulated**				
KEGG_FOCAL_ADHESION	188	0.57	1.57	3055
KEGG_ECM_RECEPTOR_INTERACTION	81	0.64	1.56	2936
KEGG_VEGF_SIGNALING_PATHWAY	71	0.43	1.53	2721
KEGG_HYPERTROPHIC_CARDIOMYOPATHY_HCM	82	0.56	1.50	3558
KEGG_COMPLEMENT_AND_COAGULATION_CASCADES	66	0.63	1.48	2195
KEGG_VASCULAR_SMOOTH_MUSCLE_CONTRACTION	110	0.52	1.45	2423
**Downregulated**				
KEGG_PEROXISOME	72	-0.53	-0.52	5159
KEGG_GLYCOSPHINGOLIPID_BIOSYNTHESIS_LACTO_AND_NEOLACTO_SERIES	26	-0.63	-1.47	1470
KEGG_PROPANOATE_METABOLISM	31	-0.56	-1.43	3648
KEGG_MATURITY_ONSET_DIABETES_OF_THE_YOUNG	19	-0.73	-1.42	1248

ES, enrichment score; NES, normalized enrichment score.

“KEGG_FOCAL_ADHESION”, “KEGG _ECM_RECEPTOR_INTERACTION” 

“KEGG_VEGF_SIGNALING_PATHWAY”, and “KEGG_HYPERTROPHIC_CARDIOMYOPATHY_HCM”, and so on.

**Figure 7:**
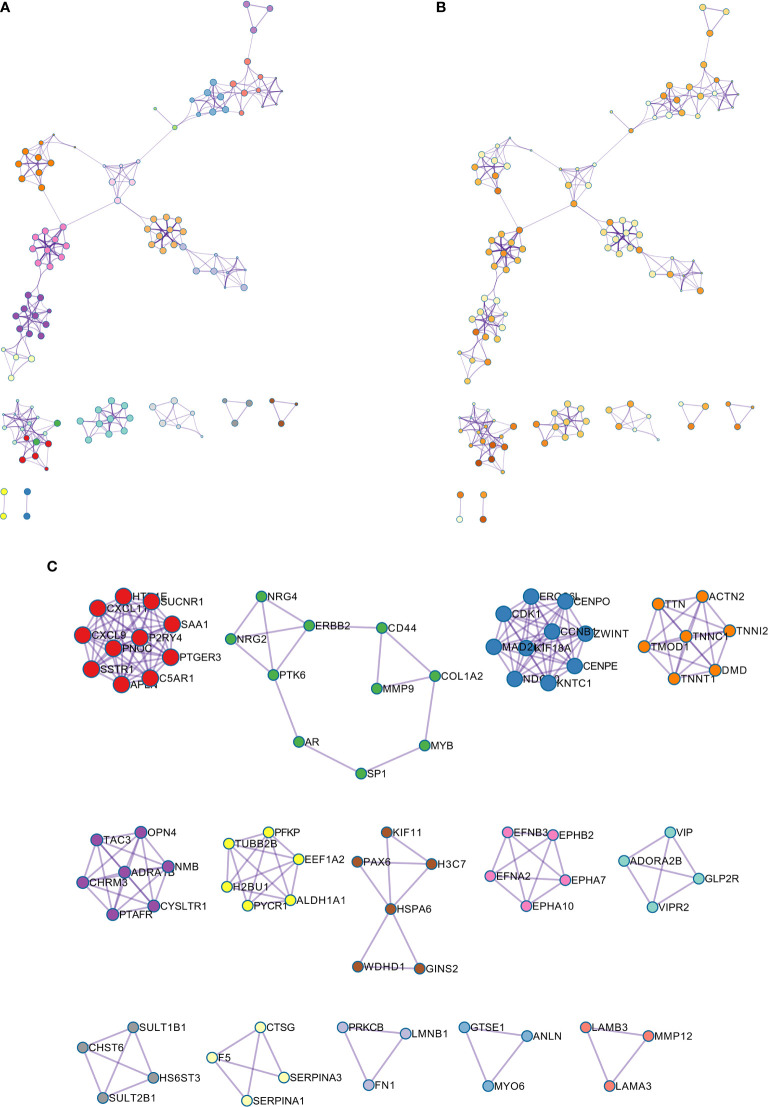
**(A)** Colored by cluster ID, where nodes that share the same cluster ID are typically close to each other; **(B)** colored by p-value, where terms containing more genes tend to have a more significant p-value. **(C)** Protein-protein interaction network and MCODE components identified in the gene lists.

The GO enriched terms of genes functions are shown in **Figure 6G**. To further explore the relationships between the terms, we selected items with the best p value from 20 clusters, each cluster did not exceed 15 items, and a total of not more than 250 items. We used Metascape for visual analysis, where each node represented a term, and was colored first by its cluster ID (**Figure 7A**). Then, these terms were analyzed by their p values (**Figure 7B**).

### The Analysis of Hub Genes

**Figure 8:**
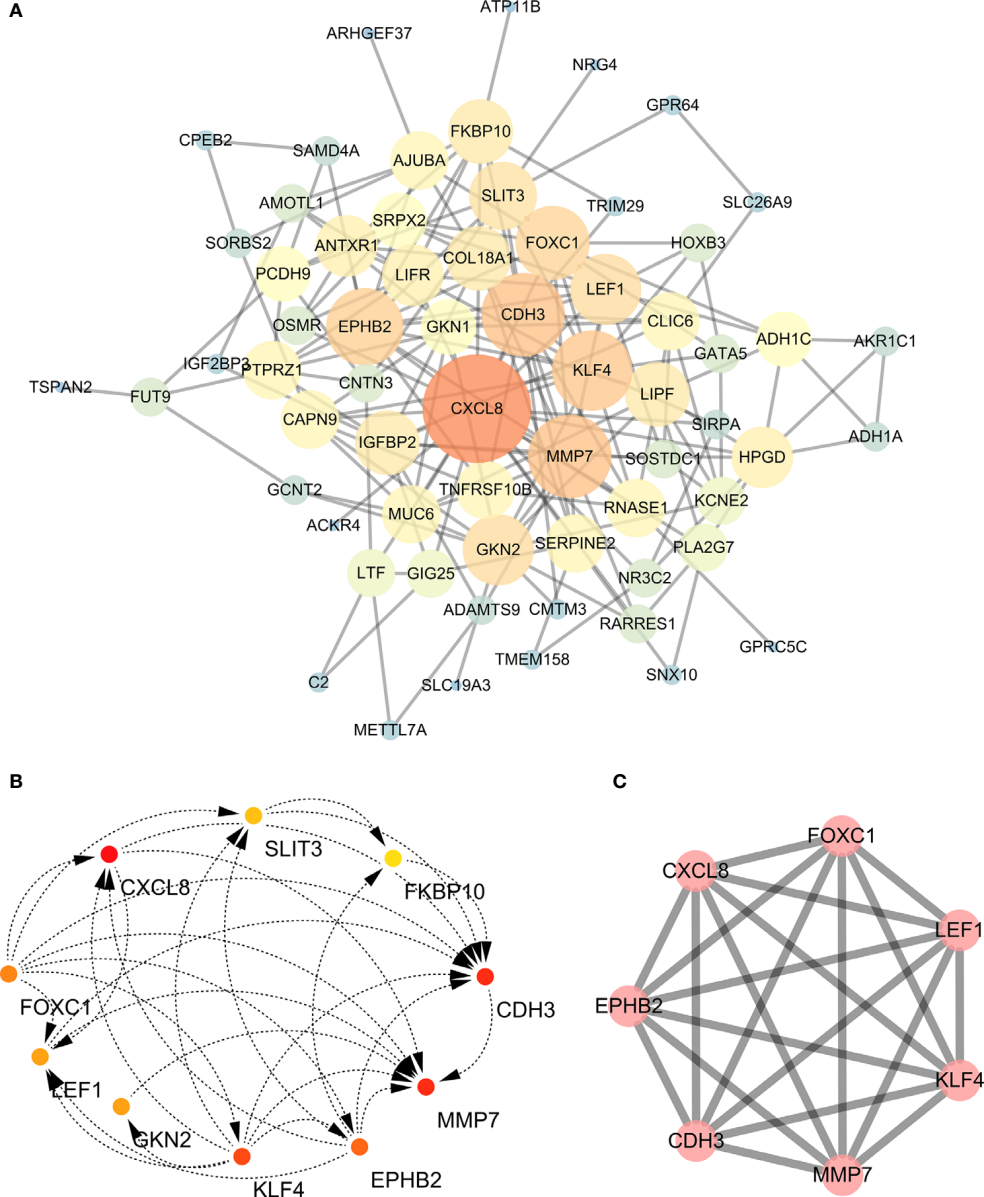
**(A)** The protein-protein interaction (PPI) network of differentially expressed genes (DEGs). **(B, C)** The hub genes were identified from the PPI network and MCODE.

A PPI network was constructed for the common differentially expressed genes in the four datasets (**Figure 8A**) and major nodes were analyzed (**Figure 7C**). The hub genes were obtained by Cytoscape analysis (**Figure 8B**). MCODE analysis was used to identify the most important modules (**Figure 8C**). The threshold was set as degree of ≥10 to obtain 10 hub genes. These genes may serve as important biomarkers and need to be further validated.

### Pathological Analysis and Survival Curve of Hub Genes

**Figure 9:**
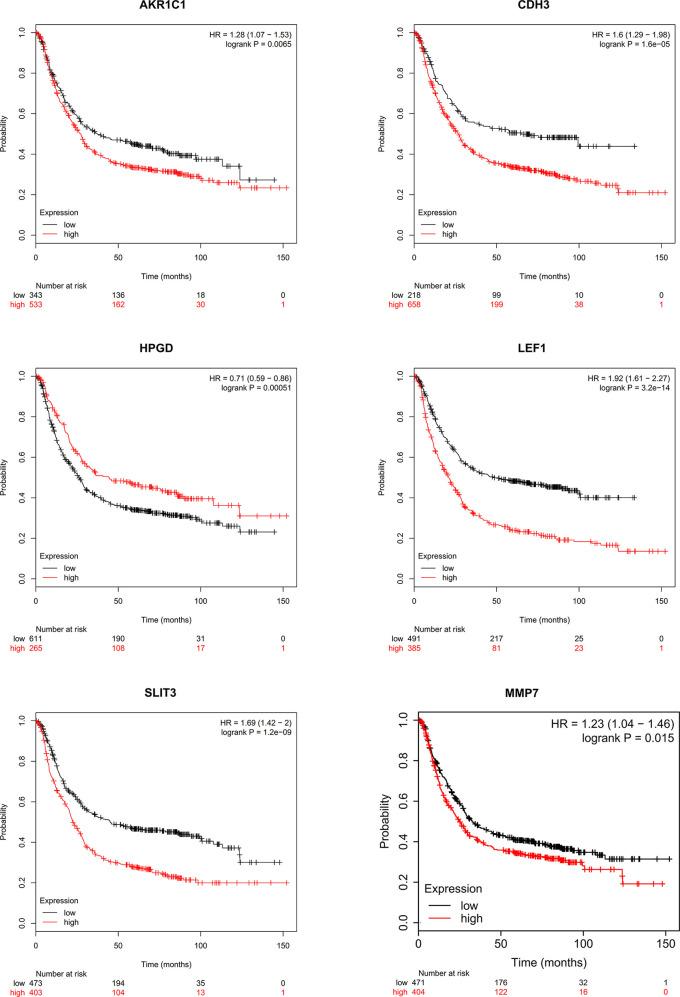
Survival analysis of hub genes.

**Figure 10:**
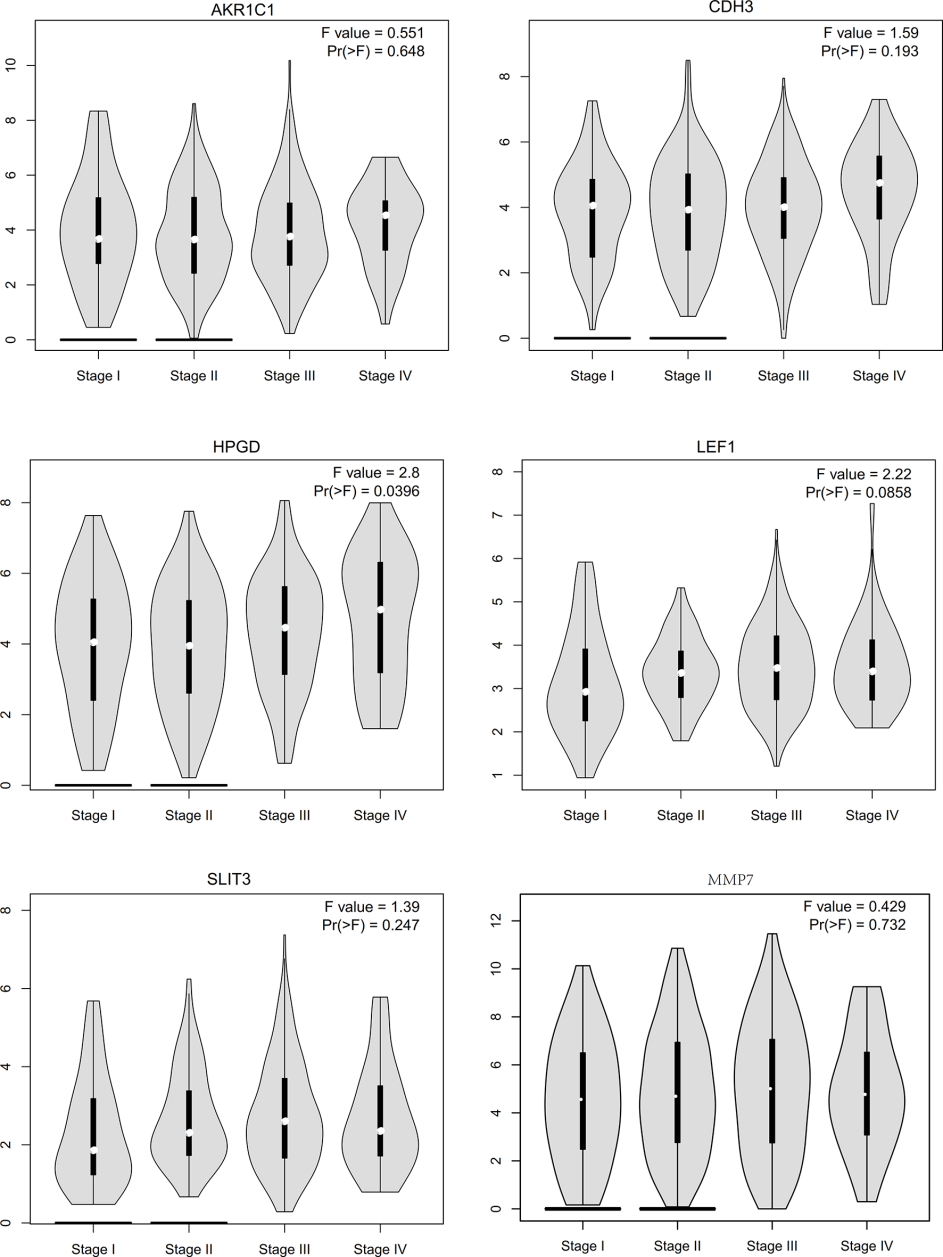
The stage plots of hub genes were generated by GEPIA.

We conducted a literature survey on the hub genes, and performed survival analysis for gastric cancer biomarkers with little or no research in the field. We found that aldo-keto reductase family 1 member C1 (AKR1C1), CDH3, LEF1, SLIT3, MMP7, and 15-hydroxyprostaglandin dehydrogenase (HPGD) genes were significantly correlated with prognosis (**Figure 9**). Subsequently, we conducted a pathological staging study on hub genes (**Figure 10**), and found that HPGD had a significant difference in the progression of gastric cancer (stage3-4, P<0.05), suggesting that it could be a new biomarker for the diagnosis and prevention of gastric cancer metastasis.

### Verification of the Expression of MMP7, CDH3, and LEF1

**Figure 11:**
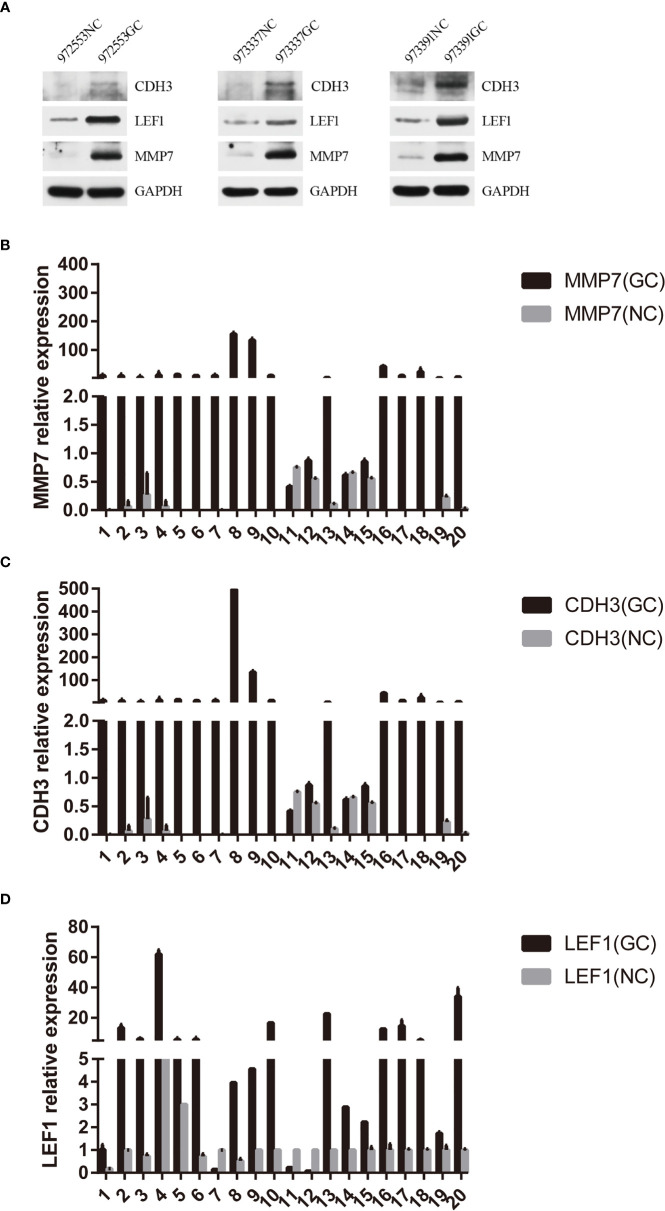
Hub gene validation. **(A)** Western blotting expression of CDH3, LEF1, and MMP7 in the normal control (NC) and gastric cancer (GC) groups. **(B)** Relative expression of MMP7 by RT-qPCR analysis. P<0.05, compared with control. **(C)** Relative expression of CDH3 by RT-qPCR analysis. P<0.05, compared with control. **(D)** Relative expression of LEF1 by RT-qPCR analysis. P<0.05, compared with control.

Based on the above bioinformatics analysis, three genes (MMP7, CDH3, LEF1) were markedly upregulated in gastric cancer samples and there was a good interaction among the three genes. The results of western blotting showed that the relative expression level of CDH3, LEF1, and MMP7 was significantly higher in gastric samples, compared with the normal groups (p<0.05) (**Figure 11A**). And MMP7, CDH3, and LEF1 levels were verified from the relative expression level by RT-qPCR (**Figures 11B–D**). The result demonstrated that MMP7, CDH3, and LEF1 might be identified as biomarkers for gastric cancer. The animal experiment also showed that these three genes were significantly increased in the gastric cancer group by RT-qPCR (**Supplementary Figure 1**).

### Strong Associations Between the Relative Expression of the Three Gene and Gastric Cancer

**Figure 12:**
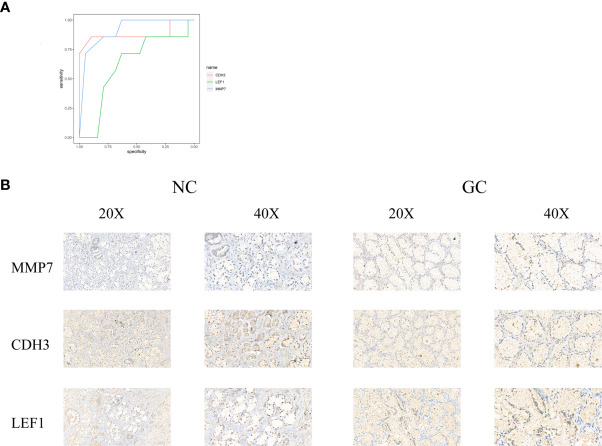
**(A)** The ROC of three genes for gastric cancer. **(B)** The detection of MMP7, CDH3, and LEF1 in the gastric tissues by immunohistochemical staining with their own antibodies. (200X, 400X).

**Figure 13:**
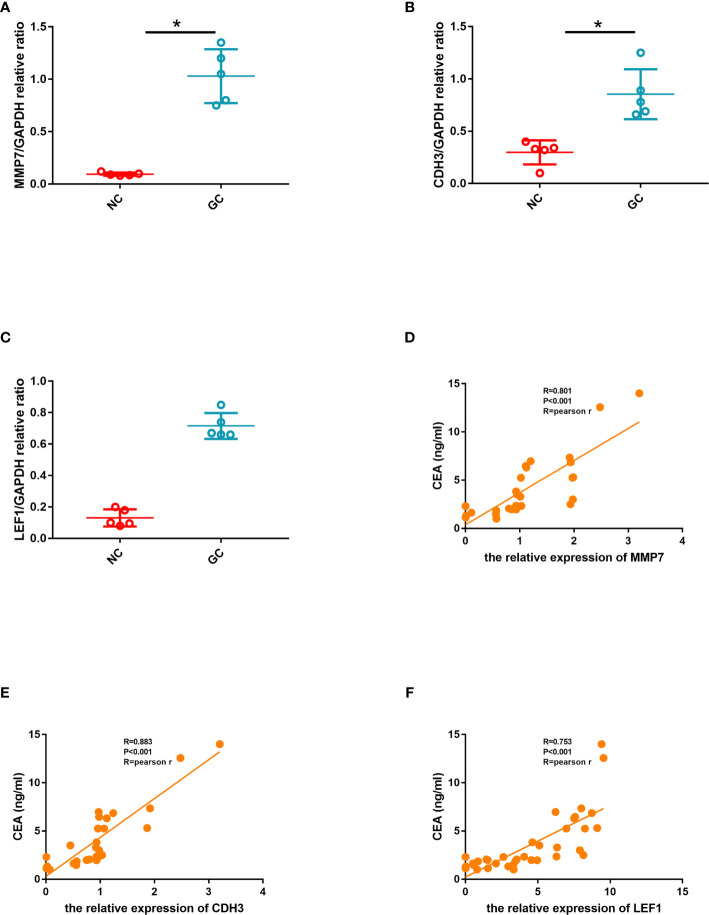
**(A)** Quantitative comparison of MMP7 expression between the two groups. **(B)** Quantitative comparison of CDH3 expression between the two groups. **(C)** Quantitative comparison of LEF1 expression between the two groups. **(D)** The linear correlation between CEA and the relative expression of MMP7. **(E)** The linear correlation between CEA and the relative expression of CDH3. **(F)** The linear correlation between CEA and the relative expression of LEF1.

ROC curves were constructed to determine the effect of the three genes’ expression on gastric cancer, and the AUC was used to determine the degree of confidence: CDH3 (AUC = 0.800, P<0.05, 95% CI =0.857-0.895), LEF1 (AUC=0.620, P<0.05, 95%CI=0.632-0.714), and MMP7 (AUC=0.914, P<0.05, 95%CI=0.714-0.947) (**Figure 12A**). Immunohistochemical results showed that the expression level of MMP7, CDH3, and LEF1 in gastric cancer tissues was higher than in the control group (**Figure 12B**). A western blotting experiment was carried out, and the results showed that the relative expression levels of MMP7, CDH3, and LEF1 in gastric cancer tissues were increased compared with the control group (**Figures 13A–C**). These results suggest that MMP7, CDH3, and LEF1 may be biomarkers for gastric cancer.

In order to further explore the correlation between hub genes and CEA which indicates the severity of gastric cancer, we calculated the linear correlation between the hub genes and CEA. CEA was positively associated with the relative expression of MMP7 (Pearson Rho=0.801, P<0.001) (**Figure 13D**). CEA was positively associated with the relative expression of CDH3 (Pearson Rho=0.883, P<0.001) (**Figure 13E**). CEA was positively associated with the relative expression of LEF1 (Pearson Rho=0.753, P<0.001) (**Figure 13F**).

### Association Between Three Genes and Gastric Cancer by Pearson’s Correlation Test and Univariate Linear Regression

Pearson’s correlation coefficient displayed that gastric cancer outcome was significantly correlated with the expression of MMP7, CDH3, and LEF1 (p<0.05). Gastric cancer remained related to MMP7, CDH3, and LEF1 (p<0.05) in the univariate linear regression model (**Table 4**).

**Table 4:** The correlation and linear regression analysis between GC and relevant gene expression.

Gene symbol	GC			
	Pearson’s correlation coefficient		Univariate linear regression	
	ρ^a^	p-value	β^b^	p-value
**MMP7**	0.359	0.023*	0.021	0.022*
**CDH3**	0.265	0.025*	-0.05	0.025*
**LEF1**	1	<0.001*	0.013	0.029*

a Pearson’s correlation coefficient between GC and relevant characteristics; ρ, Pearson’s correlation coefficient.

b Univariate linear regression analysis, β, parameter estimate; GC: gastric cancer.

*Significant variables: P<0.05.

### The Neural Network Prediction Model and High-Risk Warning Range of Gastric Cancer

**Figure 14:**
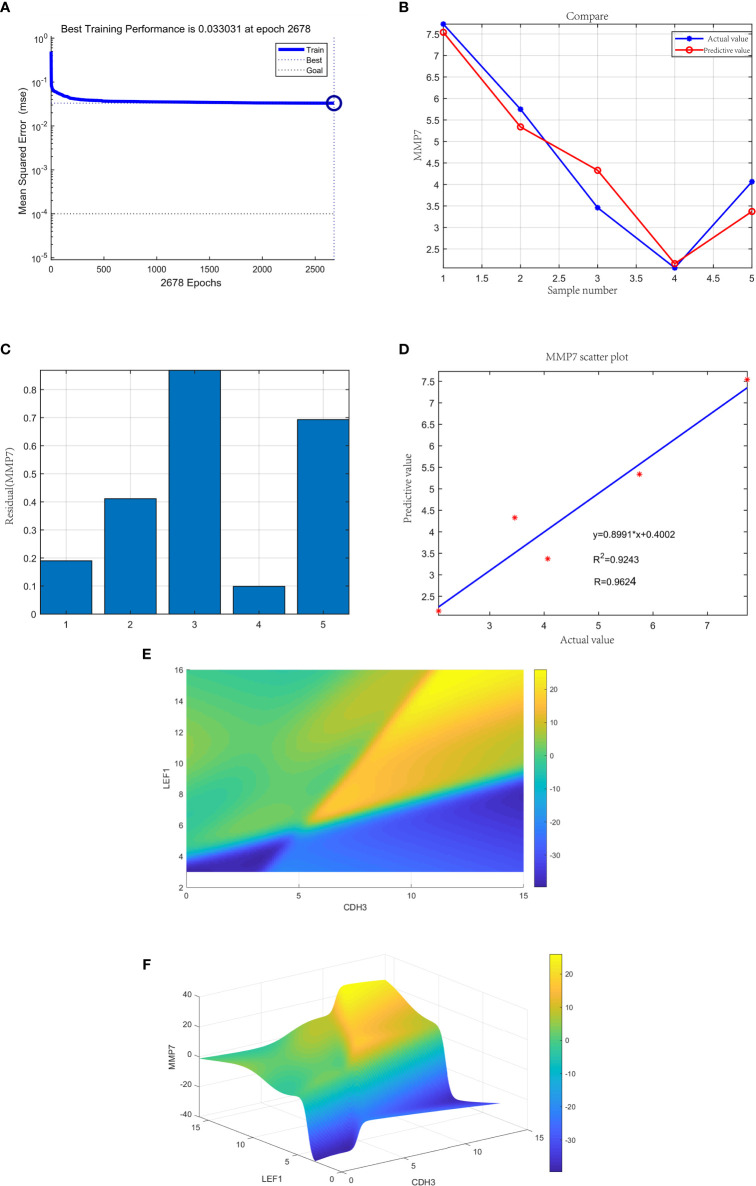
**(A)** The neural network prediction model of gastric cancer. The best training performance was 0.033031 at epoch 2678. **(B)** The predicted value of the data was verified against the actual value. **(C)** Residual plot of difference between actual and predicted values. **(D)** The final training model of neural network prediction model, and the relativity was 0.9624. **(E)** The high-risk warning range of gastric cancer at the level of the planform. **(F)** The high-risk warning range of gastric cancer at the level of the three-dimensional stereogram. The color represents MMP7 expression: “yellow” represents “high” and “blue” represents “low”.

The neural network model was trained with 35 gastric samples as the training set, and 5 gastric samples as the validation set. After training, the neural network prediction model achieved the best results. The best training performance was 0.033031 at epoch 2678 (**Figure 14A**). The difference between the predicted value and the true value was very small (**Figure 14B**), and the residual plot showed the same result (**Figure 14C**). The predicted value was fitted to the true value, and the correlation coefficient was 0.9624 (**Figure 14D**). In summary, the expression of the three genes may be joint predictors of gastric cancer.

Through the cubic spline interpolation algorithm, we found that the high-risk warning indicator of gastric cancer was 8<CDH3<15 and 10<expression of LEF1<16 (**Figure 14E**). Therefore, the 3D stereogram can better represent the early warning range (**Figure 14F**).

## Discussion

Gastric cancer is one of the diseases with the highest mortality rate and has a high incidence in China. Recently, although many comprehensive treatments have been used to improve the efficacy, the 5-year survival rate of gastric cancer is still only 20%-30% (19). Actively exploring relevant biomarkers, early diagnosis, reasonable assessment of their prognosis, and timely intervention are of great significance for clinical treatment. With the advancement of molecular biology research methods, new types of prognostic biomarkers have emerged in an endless stream, making prognostic evaluation more objective (20).

In previous studies, CD44v9 was found to be highly expressed in mouse gastric cancer proliferating cells, and CD44v9 positive immune expression can be considered as a prognostic indicator of early gastric cancer, but not as a prognostic indicator of advanced gastric cancer (21). Therefore, CD44v9 can be considered as a prognostic biomarker for early gastric cancer (22). Epidermal growth factor receptor 1 (HER1) is the growth factor of epidermal factor receptor (EGFR) gene coding, and is one of the four members of the human epidermal growth factor receptor family in the receptor tyrosine kinase superfamily (23). It works by binding specific ligands including epidermal growth factor and transforming growth factor-α which are then are activated. EGFR over expression is not only a prognostic indicator in gastric cancer, but also can be used as a basis for personalized treatment. HER1 can also be used as a therapeutic target for gastric cancer (24). HER2 is encoded by ERBB2 and is a member of the HER family. Unlike other members of the HER family, HER2 does not contain sites and signals that bind to other heterodimer ligands (25). In gastric cancer, 4%-7% of tumors were found to have ERBB2 amplification or HER2 overexpression. Studies have shown that the expansion of ERBB2 is associated with a poor prognosis of gastric cancer. Therefore, the identification and research of potential biomarkers are crucial for early diagnosis and prognosis (26).

By analyzing four microarray datasets in the current study, we found the differences between gastric cancer and normal tissues adjacent to the cancer. The four datasets contained a total of 70 DEGs, and their interactions were explored through KEGG and GO analysis. DEGs are mainly concentrated in angiogenesis, the oncostatin-M-mediated signaling pathway, cytokine-cytokine receptor interaction, and glycolysis/gluconeogenesis. Studies have reported that angiogenesis and glycolysis have an important influence on the occurrence and progression of gastric cancer (27, 28). In addition, recent studies have found that cytokine-cytokine receptor interactions play a significant part in the development of gastric cancer (29). The expression of nerve growth factor receptor p75 (p75NTR) in gastric cancer cells is significantly lower than in adjacent tissues, suggesting that p75NTR may play a significant role in gastric cancer metastasis (30, 31). The results of this paper are consistent with the above studies.

By analyzing public and private datasets, we found that MMP7 expression is highly expressed in gastric tissues. Matrix metalloproteinase is a protease secreted by endothelial cells, fibroblasts, and smooth muscle cells, which can degrade all extracellular matrix proteins (32). MMP-7 degrades IGFBP-3 by interacting with insulin-like growth factor binding protein 3, so that insulin-like growth factor 1 exerts an anti-apoptosis effect and promotes cell mitosis (33). MMP-7 can also degrade the Fas ligand on the cell membrane, inhibit Fas-mediated apoptosis, and promote tumor growth. The encoded preproprotein is proteolytically processed to generate the mature protease (34). The gene is highly expressed in digestive, urinary, breast, and lung cancer tissues (35, 36).

In a case-control study, Fu et al. investigated whether the MMP7 promoter (A-181G and C-153T) polymorphism genotype was a risk factor for gastric cancer in Taiwan. The GG genotype of MMP7 A-181G was identified as a risk factor for gastric cancer (37).

Li et al. found that MMP7 induced T-DM1 resistance and resulted in a poor prognosis of gastric adenocarcinoma in a DKK1-dependent manner. Exogenous overexpression of MMP7 promoted T-DM1 resistance and tumor growth, while MMP7 knockdown was associated with the opposite phenotype. Moreover, DKK1 knockout can lead to decreased expression of MMP7 and resistance to T-DM1 (38). All these results indicate that MMP7 is a very important biomarker for gastric cancer. However, since gastric cancer is not caused by a single gene, it often leads to multi-gene changes. Looking for a promising combination of multiple genes to predict gastric cancer will be more conducive to the early diagnosis of gastric cancer.

CDH3 can bind to cells and the extracellular matrix (39). CDH3 is highly expressed in cancer tissues such as colorectal cancer, thyroid cancer, and pancreatic cancer, but the study of CDH3 in gastric cancer is not completely clear. Hibi K et al. found that CDH3 gene demethylation occurred in 69% of GC patients, and CDH3 demethylation was significantly associated with increased TNM staging (39). CDH3 plays a role in cell-cell adhesion in epithelial tissue and plays a key role in maintaining tissue integrity and morphology. Alterations of CDH3 can lead to tissue disruption, cell dedifferentiation, increased tumor cell aggressiveness, and ultimately metastasis. Our research found that CDH3 is highly expressed in gastric cancer tissues, and it is included in the neural network model for prediction (40). In the future, it may be used as a new biomarker for the early diagnosis of gastric cancer or the prognostic judgment of its progression.

LEF1, located on the q23-q25 region of human chromosome 4, is the downstream nuclear transcription factor of the Wnt/β-catenin signaling pathway (41). LEF1 binds to β-catenin to regulate the expression of downstream molecules, thereby regulating the signal pathway (42). LEF1 is highly expressed in acute myelogenous leukemia, prostate cancer, small lymphocytic lymphoma, and other cancers (43, 44). microRNA-6852 has been shown to inhibit the progression of stomach and colorectal cancer. Wang et al. found that the expression of LEF1 was negatively correlated with the expression of miR-6852. miR-6852 inhibited proliferation, migration, and invasion of glioma cells by inhibiting LEF1 (45). In our study, LEF1 was found to predict gastric cancer jointly with CDH3 and MMP7, which may be used as a diagnostic marker for gastric cancer in the future. In view of the complexity of the pathogenesis factors of gastric cancer, we should focus on multiple targets to accurately judge the diagnosis and prognosis of the disease, which is better than single gene predictions.

### Limitations

Despite the rigorous bioinformatics analysis performed in this study, some limitation are still present. The small sample size of our study may cause some bias in the results.

### Future Directions

In subsequent studies, we will conduct MMP7, CDH3, and LEF1 validation and molecular mechanism studies at the animal level. Multi-center randomized controlled clinical trials should be conducted and more subjects recruited to verify the role of the levels of the three genes expressed in the progression of gastric cancer.

The bioinformatics analysis was used to predict the function and expression of 10 hub genes, providing guidance for subsequent research and exploration. This study shows that MMP7, CDH3, and LEF1 are highly expressed in gastric cancer tissues. Also, we found a correlation between the three by constructing a neural network model, and the disease status could be judged through the high-risk early warning range.

## Conclusion

The bioinformatics analysis was used to predict the function and expression of 10 hub genes, providing a possible mechanism for subsequent research and exploration. This study showed that MMP7, CDH3, and LEF1 are highly expressed in gastric cancer tissues. We selected CDH3, LEF1, and MMP7 as candidate biomarkers to construct a back propagation neural network model from hub genes, which may be helpful for the early diagnosis of cancer through the high-risk early warning range.

## Data Availability Statement

The datasets presented in this study can be found in online repositories. The names of the repository/repositories and accession number(s) can be found in the article/**Supplementary Material**.

## Ethics Statement

All experiments were approved by the Ethics Committee of the Fourth Hospital of Hebei Medical University. And written informed consent was obtained from all the patients.

## Author Contributions

The bioinformatics data were analyzed by M-jS. M-jS was also a major contributor in writing the manuscript. L-bM and Y-mZ proofread and submitted the article. L-bM, DK, Y-bL and PG designed the draft of the research process and offered funding for the experiment. All authors contributed to the article and approved the submitted version.

## Funding

The present study was supported by the Youth Science and Technology Project of Health and the Health Commission of Hebei Province (Hebei, China; grant nos. 20170732, 20180558).

## Conflict of Interest

The authors declare that the research was conducted in the absence of any commercial or financial relationships that could be construed as a potential conflict of interest.

## Publisher’s Note

All claims expressed in this article are solely those of the authors and do not necessarily represent those of their affiliated organizations, or those of the publisher, the editors and the reviewers. Any product that may be evaluated in this article, or claim that may be made by its manufacturer, is not guaranteed or endorsed by the publisher.

## Supplementary Material

Supplementary Figure 1 **(A)** Relative expression of MMP7 in mouse model of carcinoma in situ by RT-qPCR analysis. P<0.001, compared with control. **(B)** Relative expression of CDH3 in mouse model of carcinoma in situ by RT-qPCR analysis. P<0.001, compared with control. **(C)** Relative expression of LEF1 in mouse model of carcinoma in situ by RT-qPCR analysis. P<0.001, compared with control.

## Abbreviations

GC, gastric cancer; GEO, Gene Expression Omnibus; PPI, protein-protein interaction; GO, Gene Ontology; KEGG, Kyoto Encyclopedia of Genes and Genomes; CEA, carcinoembryonic antigen; CA19-9, carbohydrate antigen 19-9; DEGs, differentially expressed genes; WGCNA, weighted gene co-expression network analysis; GSEA, gene set enrichment analysis; CDH3, cadherin-3; LEF1, lymphoid enhancer-binding factor 1; MMP7, matrix metallopeptidase 7; ROC, receiver operator characteristic; PCA, principal component analysis; BP, biological processes; CC, cell components; AKR1C1,aldo-keto reductase family 1 member C1; HPGD,15-hydroxyprostaglandin dehydrogenase; HER1, epidermal growth factor receptor 1; EGFR, growth of epidermal factor receptor; p75NTR, nerve growth factor receptor p75.
