# Supplementary figures and images for: Screening and Identification of Key Biomarkers of Gastric Cancer: Three Genes Jointly Predict Gastric Cancer
Source: Front Oncol. 2021 Aug 17;11:591893. doi: 10.3389/fonc.2021.591893 (PMC8416116; doi:10.3389/fonc.2021.591893)

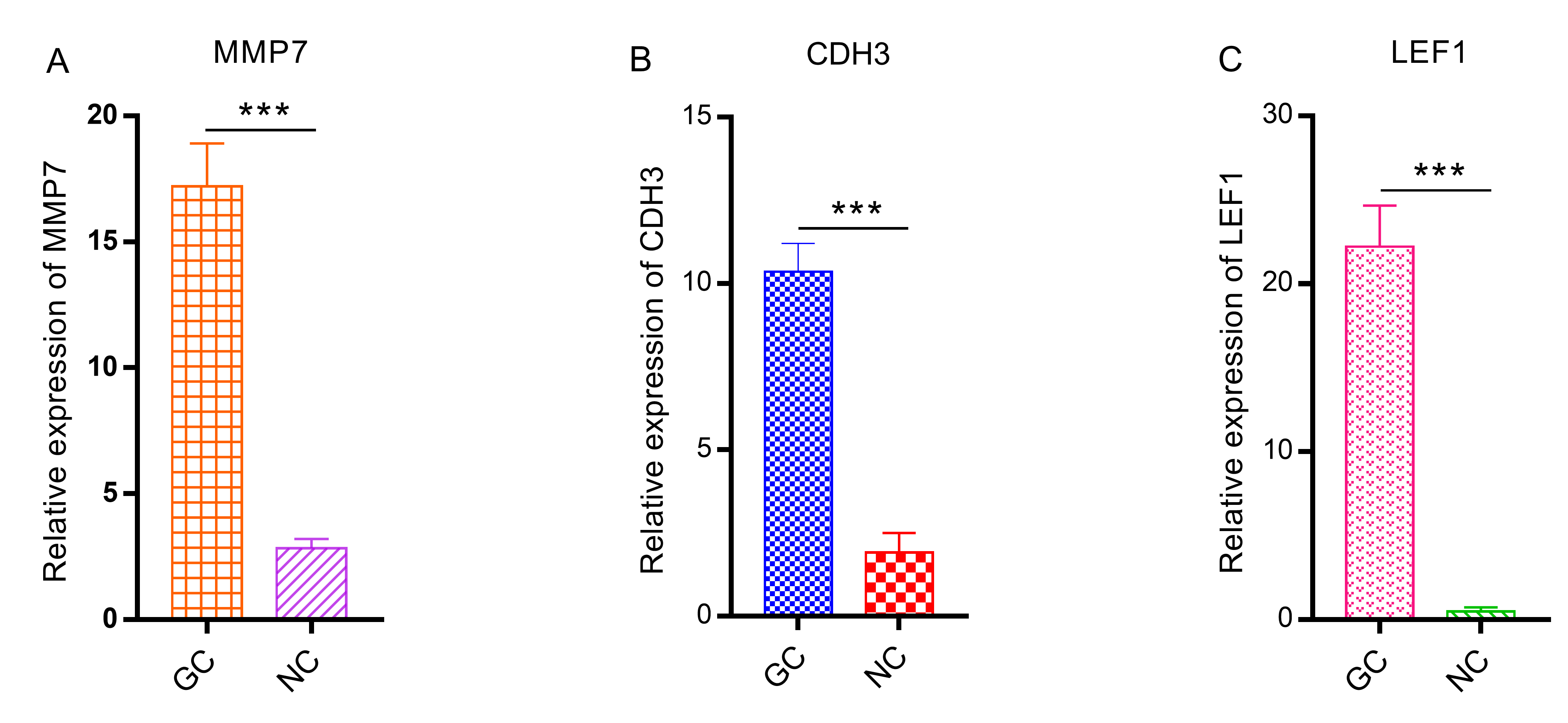

Supplement: Supplementary Figure 1 — (A) Relative expression of MMP7 in mouse model of carcinoma in situ by RT-qPCR analysis. P<0.001, compared with control. (B) Relative expression of CDH3 in mouse model of carcinoma in situ by RT-qPCR analysis. P<0.001, compared with control. (C) Relative expression of LEF1 in mouse model of carcinoma in situ by RT-qPCR analysis. P<0.001, compared with control. [file Image_1.tif]
